# Molecular characterization of the re-emerging West Nile virus in avian species and equids in Israel, 2018, and pathological description of the disease

**DOI:** 10.1186/s13071-020-04399-2

**Published:** 2020-10-22

**Authors:** Gili Schvartz, Yigal Farnoushi, Asaf Berkowitz, Nir Edery, Shelly Hahn, Amir Steinman, Avishai Lublin, Oran Erster

**Affiliations:** 1 Division of Virology, Kimron Veterinary Institute, Bet Dagan, Israel; 2 Division of Avian diseases, Kimron Veterinary Institute, Bet Dagan, Israel; 3 Division of Pathology, Kimron Veterinary Institute, Bet Dagan, Israel; 4 Koret School of Veterinary Medicine, The Robert H. Smith, Faculty of Agriculture, Food and Environment, The Hebrew University of Jerusalem, 7610001 Rehovot, Israel; 5 Present Address: Central Virology Laboratory, Israel Ministry of Health, Sheba Medical Center, Ramat Gan, Israel

**Keywords:** West-nile virus, Avian species, Equids, Histopathology, Phylogenetic analysis

## Abstract

**Background:**

In this report we describe the molecular and pathological characteristics of West Nile virus (WNV) infection that occurred during the summer and fall of 2018 in avian species and equines. WNV is reported in Israel since the 1950s, with occasional outbreaks leading to significant morbidity and mortality in birds, high infection in horses and humans, and sporadic fatalities in humans.

**Methods:**

Animal and avian carcasses in a suitable condition were examined by *post-mortem* analysis. Tissue samples were examined for WNV by RT-qPCR and the viral load was quantified. Samples with sufficient material quality were further analyzed by Endpoint PCR and sequencing, which was used for phylogenetic analysis. Tissue samples from positive animals were used for culturing the virus in Vero and C6/36 cells.

**Results:**

WNV RNA was detected in one yellow-legged gull (*Larus michahellis*), two long-eared owls (*Asio otus*), two domesticated geese (*Anser anser*), one pheasant (*Phasianus colchicus*), four hooded crows (*Corvus cornix*), three horses and one donkey. Pathological and histopathological findings were characteristic of viral infection. Molecular analysis and viral load quantification showed varying degrees of infection, ranging between 70–1.4 × 10^6^ target copies per sample. Phylogenetic analysis of a 906-bp genomic segment showed that all samples belonged to Lineage 1 clade 1a, with the following partition: five samples from 2018 and one sample detected in 2016 were of Cluster 2 Eastern European, two of Cluster 2 Mediterranean and four of Cluster 4. Four of the positive samples was successfully propagated in C6/36 and Vero cell lines for further work.

**Conclusions:**

WNV is constantly circulating in wild and domesticated birds and animals in Israel, necessitating constant surveillance in birds and equines. At least three WNV strains were circulating in the suspected birds and animals examined. Quantitative analysis showed that the viral load varies significantly between different organs and tissues of the infected animals.
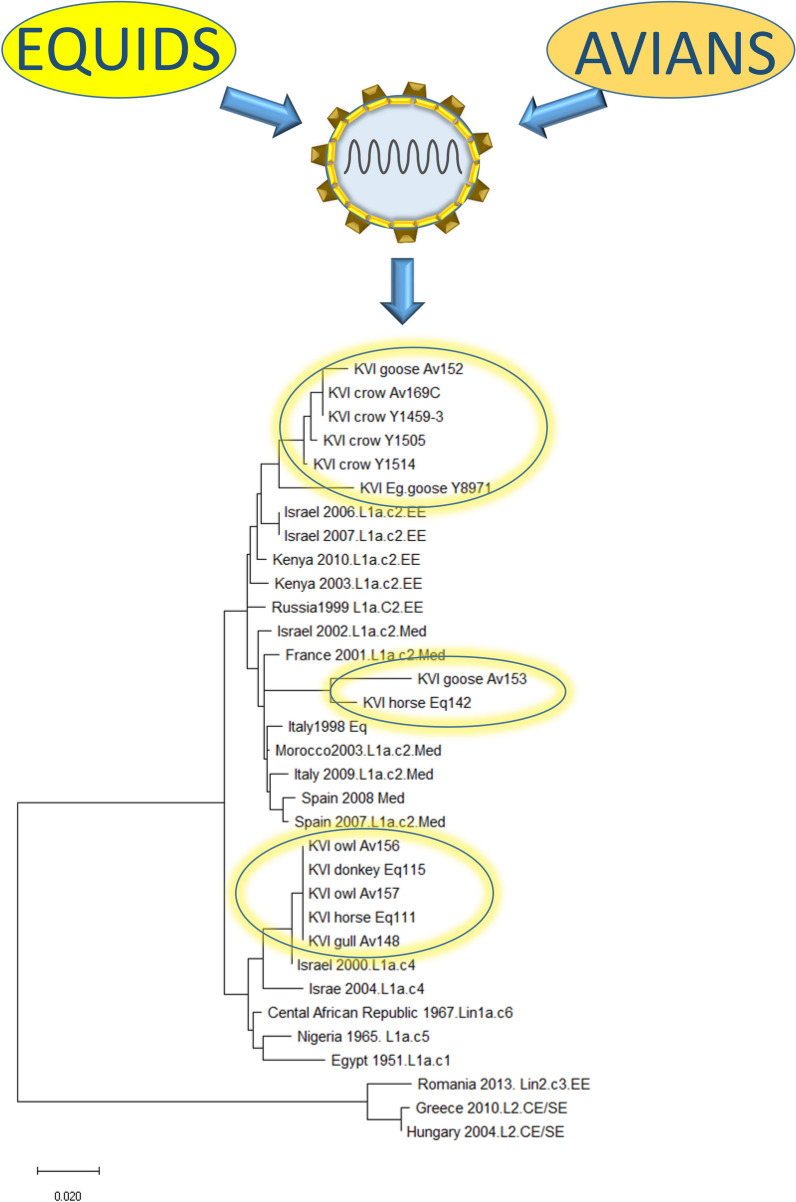

## Background

West Nile virus (WNV) is a ssRNA virus belonging to the family *Flaviviridae*, genus *Flavivirus* and is the causative agent of West Nile fever (WNF). The virus is a mosquito-transmitted pathogen affecting various species of birds, as well as horses and humans [1–3]. Over 300 species of birds have been found infected [4, 5]. While migratory and domestic avian species serve as a natural virus reservoir, humans and horses are considered dead-end hosts [1, 3, 6]. In nature, the virus is maintained in mosquito-bird-mosquito transmission cycles [7]. WNV infection in vertebrates is mostly subclinical and may cause symptoms ranging from fever, headache (in humans), malaise and other flu like symptoms, to meningoencephalitis or flaccid paralysis. Severe neuroinvasive disease in both horses and humans may lead to death and affect mostly elderly in humans, and immunocompromised individuals [1, 8]. WNV was identified in all continents except for Antarctica, where its major vector is absent [9]; therefore, it is currently recognized as one of the most widespread arboviruses [9, 10].

The first worldwide epidemic of WNV in humans was reported in Israel during the early 1950s [11], and since then it occurred sporadically, with a particularly severe outbreak during the end of the 1990s, with high morbidity and mortality of domestic geese and wild birds, especially white storks. In 2000, horses and humans were affected [8, 12, 13]. At the same time, the same WNV strain was identified in North America, the first time this virus has been detected in the Western Hemisphere [14], where more than 3000 American crows (*Corvus brachyrhynchos*) and several other birds were found dead in the New York City metropolitan area, before and concurrent with the outbreak in humans and fatal neurological disease in horses [15–17]. The largest outbreak of neurological ailments in humans in Israel occurred in 2000; it was preceded by several outbreaks in domestic geese that presented severe neurological signs with high morbidity and mortality [18]. Concomitant neurological disease related to WNV infection was reported in dozens of horses [8]. In parallel to the observed outbreak in horses and birds, multiple cases of WNV encephalitis were reported in humans in Israel: 439 patients with clinical signs, with almost 40 deaths [12]. Currently, WNV is considered endemic in Israel, as evident from serological studies, mosquito surveillance and diagnosis in human patients [19–21]. The majority of clinical cases among humans and horses occurred in the coastal plain [8, 20], with outbreaks characterized by increased pathogenicity towards wild birds, which were once considered non-susceptible and now may suffer high mortalities [9].

Diagnosis of WNV is based on observation of typical clinical signs and on several laboratory methods: virus isolation, molecular detection of the viral RNA (RT-PCR and RT-qPCR), and serological assays, such as ELISA, virus neutralization test (VNT) of two consecutives samples. In addition to ELISA-based tests aimed to determine exposure to the virus, several molecular tests were developed and are currently used to detect and classify WNV, based on its RNA sequence [22–24].

Genetic analysis of WNV obtained from mosquitoes in Israel between 2000 and 2014 attributed the majority of the samples to Lineage 1 clade 1a, Clusters 2 and 4, while during 2009 and 2010, samples belonging to Lineage 2 were also identified [3]. Analysis of samples with mosquito and human origin during 2015, attributed all samples to Lineage 1 clade 1a and Cluster 2, where the mosquito-derived samples were partitioned between the Mediterranean and Eastern European subtype, but all of the human-derived samples were from the Mediterranean subtype [25]. Genetic studies of WNV samples from animals were conducted following the 1999–2000 outbreak [8, 18]. However, recent studies on WNV infection of animals focused on serology and not on molecular analyses [10, 26].

In this report, we describe the molecular detection and characterization of WNV infection in birds and horses that were diagnosed at the Kimron Veterinary Institute (KVI), Bet Dagan, Israel, during and 2018, after more than a decade in which cases in these species were only sporadic. We then discuss the possible implications of the current situation with regards to horses, as well as wild and domestic birds.

## Methods

### Sample collection

The samples which were used for this study were collected between 29 June and 30 October 2018. Most of the avian samples were submitted for *post-mortem* (PM) examination at the Division of Avian Diseases of KVI, some of them following *ante-mortem* neurological signs. The other samples were from live birds with neurological signs, from which blood samples and cloacal swabs were taken in the Israeli wildlife hospital, as part of the routine examination and treatment of hospitalized birds. One crow sample was obtained from a private veterinary clinic. Blood and urine from suspected equine clinical samples were sent by the referring veterinarians. Visceral and central nerve system (CNS) tissues of avian and equine species were harvested during PM inspection in the Avian Diseases and Pathology laboratories, respectively. Unless processed immediately, samples were kept in − 20 °C. Whole blood was frozen before processing in order to facilitate hemolysis.

### Pathological and histopathological examination

Avian and equid carcasses that were in a good PM condition were submitted for necropsy at the KVI Laboratories of Avian Diseases and Pathology, respectively. When possible, a histological examination was consequently performed.

### RNA extraction

RNA from body fluids was extracted using the Ribospin vRDII extraction kit (GeneAll, Seoul, Soiuth Korea; https://www.geneall.com/english/) according to the manufacturer’s instructions. Whole blood was defrosted and diluted 1:5 in PBS before RNA extraction. Cloacal swabs were soaked in 500 µl PBS, vortexed and incubated for 5 min, after which the suspension fraction was collected for RNA extraction. CNS and visceral tissues were homogenized in PBS in a volume ratio of 1:5, incubated on ice for 15 min, and then centrifuged at 1100×*g* for 10 min in 4 °C. Supernatant was harvested for RNA extraction using the Ribospin kit as described above.

### Quantitative and endpoint RT-PCR (qRT-PCR and PCR)

The following primer sets were used for detection of WNV RNA in the examined samples. The test recommended by the OIE (OIE terrestrial manual) was used to detect the NS2A segment as described by the authors (Eiden et al. [24]). A separate assay was designed in the present study, based on Pol-C segment (forward primer 290Fwd: 5′-GTG CTG GAT CGA TGG AGA GG-3′; reverse primer 385Rev: 5′-GTG CTG GAT CGA TGG AGA GG-3′ and Pol-C probe: 5′-CAA ACA GCG ATG AAA CAC CTT CTG A-3*'*. AgPath-ID RT-PCR mix (cat. # 4387424; Thermo Fisher Scientific, Waltham, MA, USA) was used for the qRT-PCR assays with the Bio-Rad CFX96 thermocycler. The reaction mix contained the following components: a mutant MMLV RT 1 µl; AmpliTaq Gold^®^ polymerase; 2× RT-PCR Buffer 12.5 µl; ROX™ Dye, detection enhancer 1.5 µl; PCR water 5.7 µl; forward primer 1 µl; reverse primer 1 µl; probe 0.4 µl; 5 µl RNA; and ddH_2_O to a final volume of 28 µl. The reaction conditions were as follows: RT at 45 °C for 10 min; activation at 95 °C for 10 min; 40 cycles of 95 °C for 15 s, 60 °C for 45 s. Fluorescence was read at the 60 °C step.

In order to further establish the presence of the virus and determine its genetic classification, two genomic regions were amplified and sequenced. A region spanning the N, preM, M and envelope genes, corresponding to positions 108 through 998 on GenBank sequence HM152775 (“Kunjin fragment”) [22] was amplified. Samples in which the “Kunjin fragment” was not successfully amplified were analyzed by amplification of a genome segment spanning part of the NS1 and NS2A genes (positions 2838–3736 on GenBank sequence HM152775) or part of the Capsid region (positions 240–1266 on GenBank sequence HM152775) were amplified. For the NS1-NS2A, test primers forward (5′-CAG AAC TCG CCA ACA ACA CCT TTG T-3′) and reverse (5′-CGC CAA GTG TAC CAC GTC TCC TCC-3′) were used. For the Capsid, primers forward (5′-CAC AGC AAT TGC TCC GAC CCG-3′) and reverse (5′-CTG TGT GGA GTA GTT TCC-3′) were used. cDNA was synthesized from WNV positive samples using the Verso cDNA synthesis kit (cat. # AB1453A; Thermo Fisher Scientific), according to the manufacturer’s instructions. The cDNA was then used for amplification of the desired product using DreamTaq Green mix (cat. # EP1701; Thermo Fisher Scientific). The reaction mix was as follows: DreamTaq Green mix (25 µl); H_2_O—18 µl; 1 µl of each primer (10 µM stock concentration); and cDNA (5 µl). The reaction conditions were as follows: 95 °C for 3 min; 40 cycles of 95 °C for 20 s, 60 °C (KUN reaction) or 57 °C (NS2A-long reaction) for 30 s, and 72 °C for 1 min; followed by 72 °C for 10 min and finally incubation at 4 °C. PCR products were either purified directly from the reaction mix or excised from agarose gel following electrophoresis. Product purification was performed using the GeneJET DNA purification kit (cat. # K0502; Thermo Fisher Scientific).

### Primer design and sequence analysis

Designing and preliminary primer testing, *in silico* PCR, sequencing assembly and multiple alignments were all performed using programs embedded in the Geneious 9.1.8 package (Biomatters, Aukland, New-Zealand; www.geneious.com/about/). The “KUN” (Kunjin) segments, which were used for the phylogenetic analysis were amplified using the primers described above (“Kunjin fragment” primers). Multiple alignment analysis was performed using the ClustalW program. The alignment file was used to generate the phylogenetic tree using the Maximum Likelihood method constructed with MEGA X [27].

### Virus isolation and propagation

Both mammalian (Vero) and insect (C6/36) cell lines were used to isolate and propagate WNV from infected samples. Vero cells were grown and passaged in minimal essential medium (Sigma-Aldrich, St. Lewis, MO, USA) enriched with 10% fetal bovine serum, 0.4% l-glutamine, and 1% antibiotic mix (penicillin, streptomycin and amphotericin B) at 37 °C with 5% CO_2_. C6/36 cells were cultured in L-15 medium (Gibco, Thermo Fisher Scientific, Waltham, MA, USA) enriched with 10% FBS, 0.4% l-glutamine, 1.4% HEPES and 1% antibiotic mix. Homogenates from PCR-positive field samples were diluted 1:5 in sterile PBS and filtered through 0.22 μm sterile filter (Sartorius, Goettingen Germany). Vero monolayers (25 ml^2^ flask) or C6/36 uniform cultures were infected with filtered sample diluted 1:10 after removal of medium. Flasks were incubated for 30 min (37 °C with 5% CO_2_ for Vero or 28 °C for C6/36), washed with warm medium and then filled with new medium. Flasks were observed daily for cytopathic effect, that was recorded, and aliquots of the medium were tested by qRT-PCR to confirm virus replication.

## Results

### Clinical signs and gross pathology of affected birds and equids

Out of several dozens of suspected dead birds that were examined at the KVI Laboratory of Avian Diseases between June and October 2018, 10 were diagnosed as positive for WNV infection, as detailed in Table 1. Three of these birds presented neurological signs upon hospitalization (long-eared owl no. AV156 and hooded crows nos. AV169 and 1514), all of them died a few days after admission. *Post-mortem* analysis of the yellow-legged seagull (*Larus michahellis*, labeled AV148) that was found dead, revealed mild congestion in the leptomeninges and very pale kidneys. In one of the two domestic geese (*Anser anser*, labeled AV153, Tables 1, 2) that were found dead, the lung was dark red slightly firm, oozed large amounts of foamy blood tinged fluid and sank in water, indicating pneumonia. Also, there was hepatomegaly with white streaks on the liver’s capsule (scars), and the leptomeninges were congested.

**Table 1:** Details of the examined WNV-positive avian species

Bird no.	Species	Date	Neurological signs	Intracranial haemorrhages	Location	Comments
AV148	*Larus michahellis*, yellow legged seagull	18 July 2018	Unknown	+	Tel-Aviv	Found dead
AV152	*Anser anser domesticus*, domesticated goose	18 July 2018	+	+	Burgata^a^	Found dead in petting zoo
AV153	*Anser anser domesticus*, domesticated goose	18 July 2018	Unknown	+	Burgata^a^	Found dead in petting zoo
AV156	*Asio otus*, long eared owl	18 July 2018	Unknown	+	Hadid	Hospitalized and died
AV157	*Asio otus*, long eared owl	18 July 2018	Unknown	+	Ramat Razi’el	Found dead
AV169	*Corvus cornix*, hooded crow	18 August 2018	+	Unknown	Rishon Letzion	Found neurological and died within 24 h
AV178	*Phasianus colchicus*, common pheasant	18 August 2018	Unknown	+	Burgata^a^	Found dead in petting zoo
1459	*Corvus cornix*, hooded crow	18 October 2018	−	Unknown	Tel-Aviv	Healthy bird in quarantine
1505	*Corvus cornix*, hooded crow	18 October 2018	Unknown	+	Tel-Aviv	Found dead
1514	*Corvus cornix*, hooded crow	18 October 2018	+	Unknown	Tel-Aviv	Found sick, died in hospital

^a^Same petting zoo

*Key*: +, characteristic WNF neurological signs observed; −, no characteristic WNF neurological signs observed

**Table 2:** Pathological findings and calculated viral genome copies of WNV-positive avian species

Bird no.	Species	Common name	Sequencing	Isolation	Tissues tested and calculated target copies per reaction
AV148	*Larus michahellis*	Yellow legged seagull	Yes	Yes	Cloacal swab: 5.8 × 10^6^; brain: 2.8 × 10^6^; kidney: 1.2 × 10^7^
AV152	*Anser anser*	Domesticated goose	Yes	Yes	Cloacal swab:9.7 × 10^5^; brain 3.3 × 10^5^; kidney: 4700; eye swab: 1.3 × 10^6^
AV153	*Anser anser*	Domesticated goose	Yes	Yes	Cloacal swab: 6.8 × 10^5^; brain: 1.2 × 10^7^; viscera: 6.8 × 10^5^
AV156	*Asio otus*	Long eared owl	Yes	No	Cloacal swab: 6.8 × 10^5^; brain: 2.6 × 10^4^; viscera: 4.9 × 10^7^
AV157	*Asio otus*	Long eared owl	Yes	No	Cloacal swab: 6.8 × 10^5^; brain: 5800; kidney: 360; eye swab: 50
AV169	*Corvus cornix*	Hooded crow	Yes	No	Cloacal swab: 4700; blood: 1100
AV178	*Phasianus colchicus*	Common pheasant	No	No	Brain: 4700
1459	*Corvus cornix*	Hooded crow	Yes	No	Cloacal swab: 2300
1505	*Corvus cornix*	Hooded crow	Yes	No	Brain: 4700
1514	*Corvus cornix*	Hooded crow	Yes	Yes	Brain: 9.9 × 10^7^

**Fig. 1:**
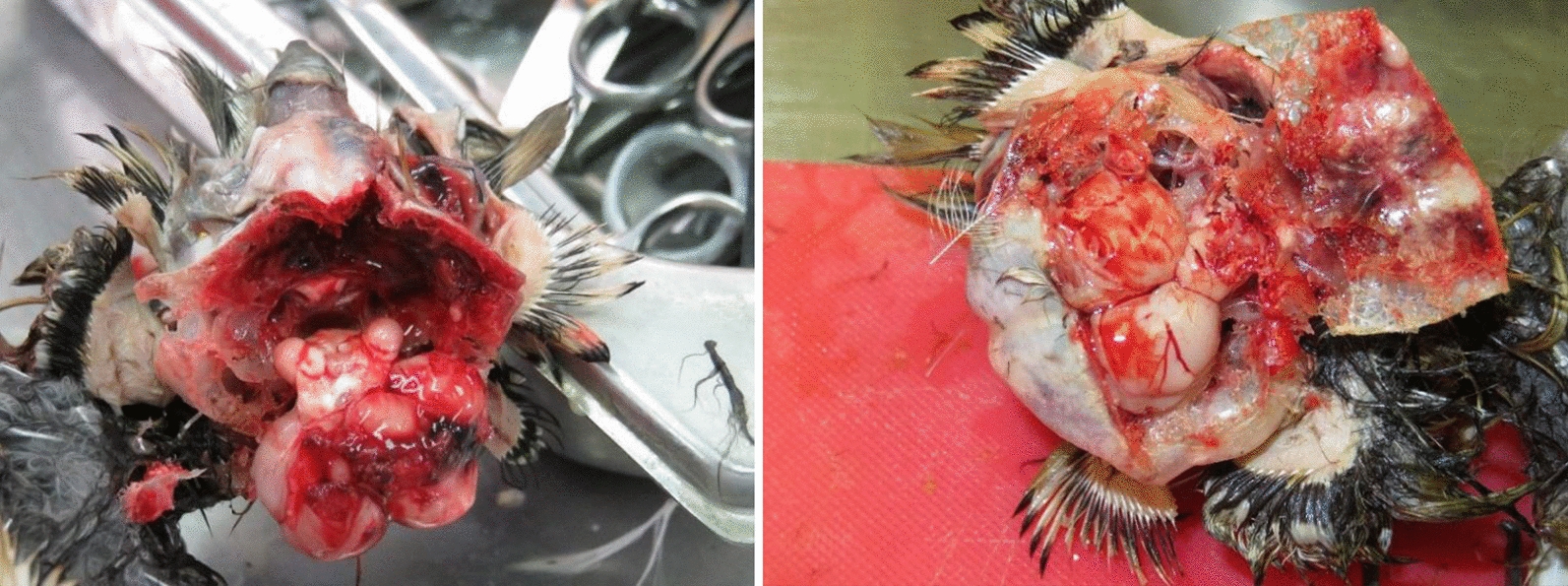
Meningeal and brain tissue hemorrhages in WNV-infected long eared owl (*Asio otus*) AV156

Both long-eared owls (*Asio otus* labelled AV156 and AV157, Tables 1, 2) which presented neurological signs, had marked leptomeningeal congestion (Fig. 1) in addition to a hematoma in the calvarium (including intraosseus hemorrhage), hemorrhage in the nasal cavity and congestion of the meninges, observed in AV157 (Tables 1, 2), which presented weakness, tremors and incoordination. In this bird, the lung was congested and there was mild splenomegaly. The dead hooded crow (*Corvus cornix*, labeled 1505, Tables 1, 2) had conspicuous intraosseus hemorrhage in the calvarium. Hooded crow no. 1459 was unsuitable for post-mortem analysis and was therefore not analyzed pathologically.

The other dead birds, a ring-necked pheasant (*Phasianus colchicus*, labeled AV178), a goose (AV152) and two hooded crows (AV169, 1514, Tables 1, 2) were not submitted for necropsy due to their poor *post-mortem* (PM) condition.

Three horses and one donkey that presented neurological clinical signs were examined as follows: a 2-year-old local breed foal (*Equus ferus caballus*, labeled Eq111, Table 3) that was euthanized due to severe neurological signs (incoordination, ataxia and recumbency) and was unresponsive to supportive treatment. A 30-year-old geriatric rescue donkey (*Equus africanus asinus*, labelled Eq115, Table 3) that suffered severe progressing neurological signs (ataxia, weakness and incoordination), deteriorated to recumbency despite supportive treatment, and was euthanized. An 11-year-old WB gelding (Eq117, Table 3) that suffered from neurological signs (muscle tremors, ataxia, incoordination and weakness) that were progressing to recumbency and puddling despite intensive supportive treatment. The horse was euthanized. Lastly, an adult rescue horse (Eq142, Table 3) with neurological recumbency was euthanized and subsequently subjected to examination.

**Table 3:** Details and calculated viral genome copies of WNF-positive equids

Animal no.	Species	Date	Location	Tissues tested and calculated target copies per reaction	Comments
Eq111 324085	Horse 1 (2-years-old)	18 June 2018	Kfar Shmu’el	Spleen: negative; brain: 2290	Euthanized
Eq115 325209	Donkey 1 (30-years-old)	18 July 2018	Gan Yoshyia	Brain: 550; spinal cord, CSF, spleen: all negative	Euthanized
Eq117 325903	Horse 2 (11-years-old)	18 July 2018	Kfar Truman	Cerebellum: 550; medulla: 4670; cervical spinal cord: 1990; thoracic spinal cord: 310; lumbar spinal cord: 680; spleen: negative; serum: negative	Euthanized
Eq142 333326	Horse 3 (20- years-old)	18 October 2018	Kfar Sirkin	NS2A probe: cerebellum: 4000; medulla: 56,400	Euthanized

### Histopathological evaluation of affected birds and equids

**Fig. 2:**
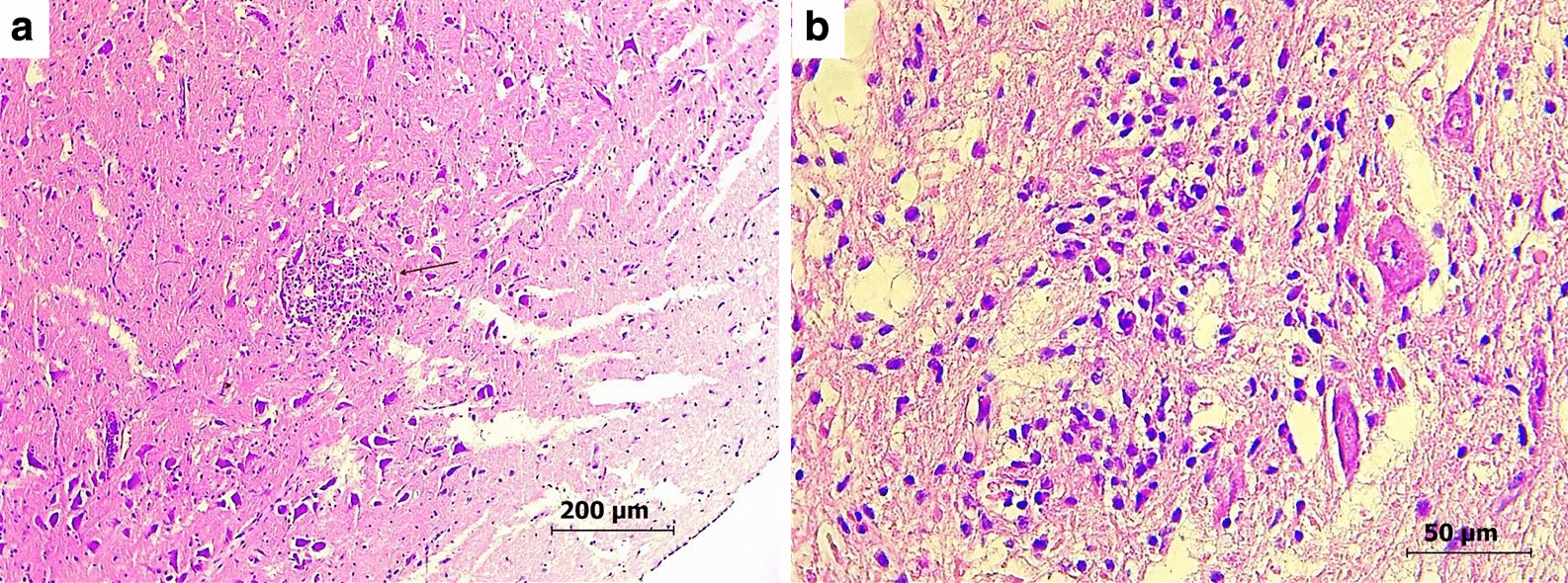
Brain histopathology of WNV-infected long-eared owl (*Asio otus*) AV156. **a** A glial nodule in the brain stem (marked by an arrow). 100× magnification. **b** A glial nodule in the brain stem with few adjacent necrotic neurons, 400× magnification

Due to poor condition of some of the birds that were screened for WNV, histopathological evaluation had been performed only on the two long-eared owls (AV156 and AV157, Fig. 2, Tables 1, 2). The main lesions in both owls were in the brain stem. These included multifocal, randomly distributed glial nodules, and admixed with small numbers of macrophages, lymphocytes and heterophils. Multifocally and randomly, the capillaries in the leptomeninges, the brain stem, and, to a lesser extent, the grey matter of the cerebrum, were congested and rarely the Virchow Rubin spaces contained very few lymphocytes and plasma cells (perivascular cuffing). There was rare neuronal necrosis (Fig. 2). Within the grey matter of the cerebrum, there was mild multifocal acute hemorrhage.

**Fig. 3:**
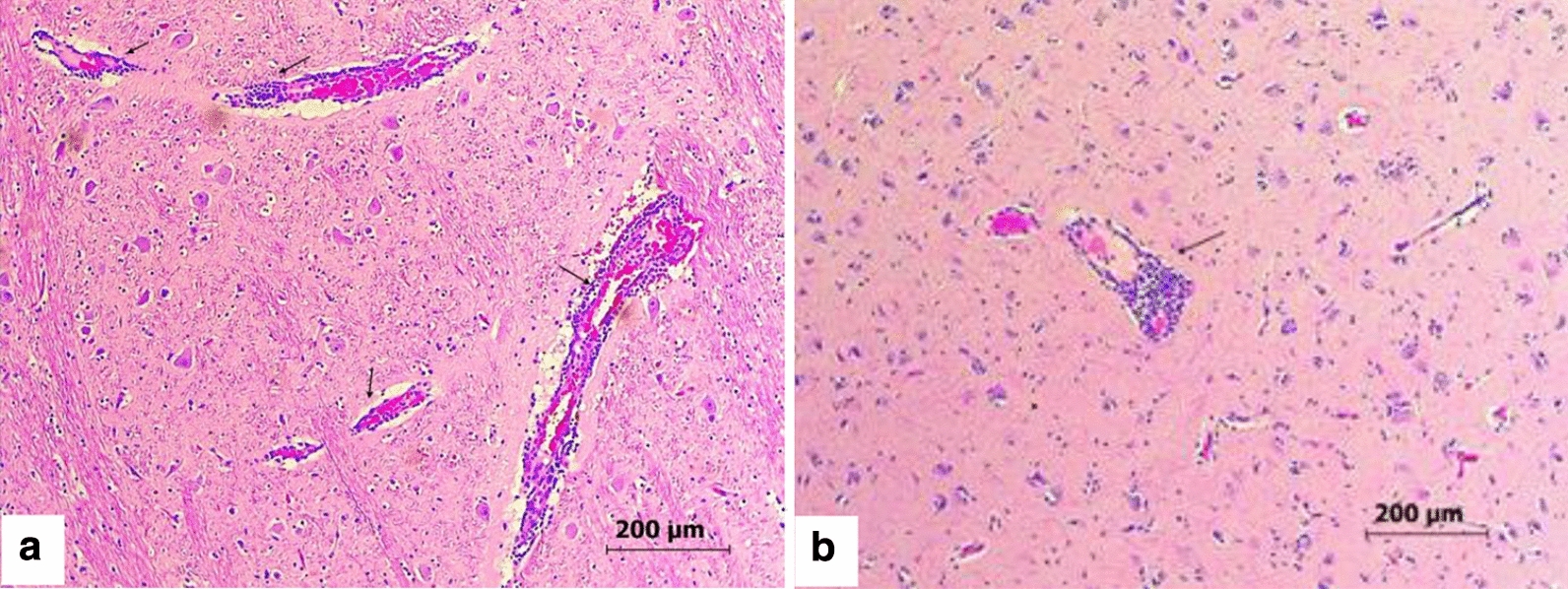
Brain histopathology of WNV-infected horses. Perivascular cuffs composed of lymphocytes and plasma cells in the brain of two horses, characteristic of viral encephalitis (marked by arrows). **a** Horse no. Eq111 (324085). **b** Horse no. Eq117 (325903). 100× magnification

The two horses that were subjected to pathological examination (Eq111 and Eq117, Table 3) presented similar lesions in the brain: the Virchow Rubin spaces surrounding capillaries were expanded by small or moderate numbers of plasma cells, lymphocytes and occasionally neutrophils (Eq117, Fig. 3a). Rarely, there were necrotic neurons with neuronophagia surrounded by glial cells. Multifocal capillaries in the brain including brain stem (especially in the white matter) were surrounded by perivascular cuffs of lymphocytes and plasma cells, accompanied by mild perivascular edema and rare extravasation. Throughout the brain stem, there were glial nodules containing few neutrophils and glial cells (Fig. 3b). Similar changes were seen in the cerebellar white matter. Rarely, there was neuronal necrosis and neuronophagia in the brain stem and cerebellum. These lesions are consistent with viral encephalitis. In general, similarly to the birds, the changes in the examined horses are most consistent with viral encephalitis. The donkey (Eq115) was sampled for PCR but was not suitable for PM examination.

### Molecular detection and isolation of WNV from equids and avian species

The presence of WNV RNA was detected by RT-qPCR in 4 equids and 8 avian species that were analyzed by KVI laboratories during 2018. Absolute target copy number calculation was performed using a standard curve for the NS2A RT-qPCR test, as follows. Serial dilution of a quantified PCR product that contains the NS2A qPCR target region were run as standards, and their Cq values were used to generate a calibration plot (Additional file 1: Figure S1). The calibration plot formula was then used to calculate the target copy number in each sample. As described above, all four equids were euthanized due to severe neurological signs, two of which were subsequently subjected to PM examination. Except for the sample derived from the horse medulla (Eq142), in which the calculated viral load was 5.6 × 10^4^ copies per sample, all samples contained viral copies of 550–4500 copies per sample, indicating low viral RNA load in the examined material, and suggesting that the viral load in the medulla of this horse was roughly tenfold to 100-fold more than in the cerebellum. The details of each animal and its test results are described in Table 3.

Avian species were either found dead or brought for analysis following hospitalization in the Israeli wild animal hospital, after dying at the hospital. Cloacal swabs were either sampled from live clinical birds or in PM analysis upon arrival to pathological examination. The results of the pathological and molecular examination are summarized in Tables 1 and 2. Examination of the brain, kidney and cloacal swab of bird AV148 (yellow-legged seagull) showed that while the brain sample contained ~ 2.8 × 10^6^ target copies per test reaction (5 µl of the eluted RNA), the swab contained ~ 5.8 × 10^6^ copies, and the kidney sample contained over 1.2 × 10^7^ copies (Table 2). The brain sample of AV157 contained ~ 5.8 × 10^3^ target copies, while the eye and cloacal swab samples contained between 50 and 6.8 × 10^5^ copies, respectively (Table 2).

**Fig. 4:**
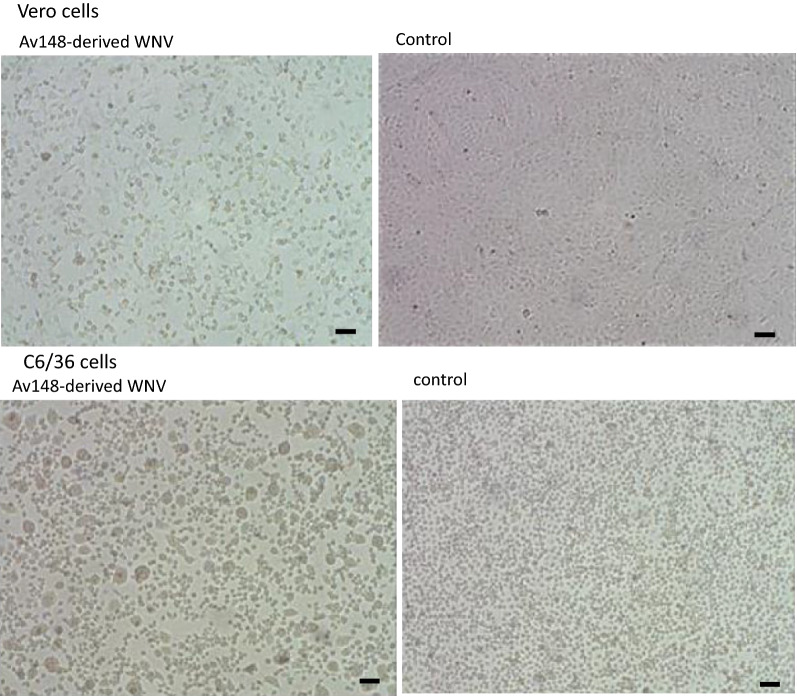
Replication of yellow-legged seagull-derived WNV in Vero and C6/36 cells. Cytopathic effect (left) was observed after one passage in both cell lines. The control cells (right) were grown under the same conditions. *Scale-bars*: 100 µM

Tissue extracts from infected avian species were prepared for virus isolation as described in the Methods section, and used to infect both Vero and C6/36 cultures. Four of the PCR positive samples (AV148, AV152, AV153 and crow no. 1514) were successfully cultured, with cytopathic effect usually observed after one passage in Vero cells and a consecutive passage in C6/36 (Fig. 4). CPE was observed in both cell lines when infected with the new isolates obtained in this study, as well as with WNV isolates obtained previously by the Kimron Institute. The virus presence was confirmed in the medium and infected cells using RT-qPCR.

### Phylogenetic analysis

**Fig. 5:**
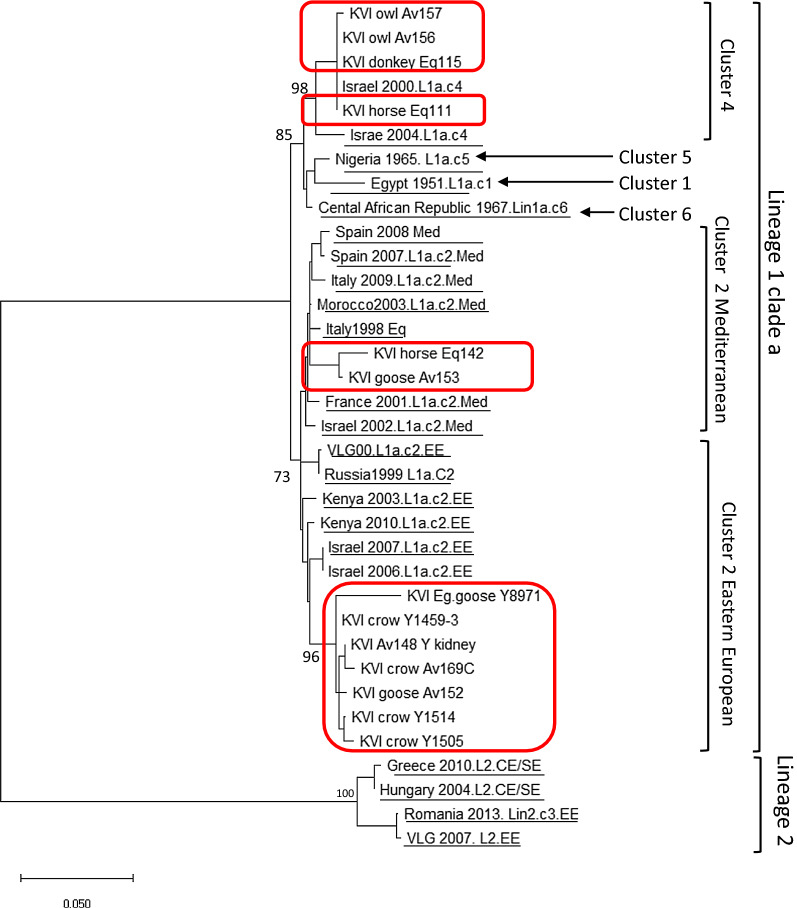
Phylogenetic analysis of West Nile viruses (WNVs) from avian and equine hosts studied in Israel during 2016 and 2018. The analysis was conducted on a nucleotide sequence of the genes encoding the capsid, pre-membrane protein, and membrane protein, using the neighbor-joining method implemented in MEGA X software. The robustness of branching pattern was tested by 1000 bootstrap replications. The rates among sites algorithm used was gamma distribution with invariant sites (G+I). The bar denotes 0.02 nucleotide substitutions per site. Lineage 1 and 2 reference strains are present with country and year of isolation. The GenBank annotated sequences are underlined and the sequences obtained in this study (during 2016 and 2018) are marked with rectangles

In order to establish the origin of the WNV that was detected in the avian and equid species, a phylogenetic analysis was performed on the 12 samples collected during 2018, on one sample collected in 2016, and 20 annotated sequences from Lineages 1 and 2. Due to variability in the sample material quality, the analysis was based on a 715-bp region spanning positions 236–950 on GenBank sequence HM152775, which was the maximal sequence length that could be obtained from all the samples, except for sample Eq142. From this sample, only 500 bp of the examined region could be sequenced from the PCR product. The resulting tree showed that all WNV samples obtained in this study during 2018 belonged to Lineage 1 clade 1a according to the following partition. Samples Eq111, Eq115, AV156 and AV157 were grouped together on Cluster 4, along with annotated samples obtained from mosquitoes in Israel during 2000 and 2004 (underlined). The bootstrap support for this branching was 85% (Fig. 5). Samples Eq142 and AV153 were located within the branch of the annotated Cluster 2 Mediterranean subtype samples (underlined), including samples that were obtained between 1998 and 2008 from Morocco, Spain, Italy, France and Israel. Lastly, samples AV8971 (obtained during 2016), AV152, AV148, AV169, AV1459, AV1505 and AV1514 were located, together with annotated Israeli samples obtained from mosquitoes in Israel during 2006 and 2007, on the Eastern European subtype branch of that cluster (Fig. 5). The bootstrap values for the branches that were generated by the two groups were lower than 80%. Nevertheless, they created a clear pattern where all the Mediterranean (Med) and Eastern European (EE) samples grouped together on two separate regions (Fig. 5). This analysis therefore suggests that during 2016–2018, at least three different subtypes from two clusters of Lineage 1 WNV, were circulating in Israel. The sequences obtained in this study are included in Additional file 1: Alignment S1.

## Discussion

The exposure of horses in Israel and the Palestinian Authority (PA) to WNV was studied relatively recently [10, 26], but actual detection of the virus in horses was last reported in the early 2000s [8]. Similarly, detection and isolation of WNV from birds was last reported in Israel at the same time [18, 28], until 2016, when the virus was successfully isolated from an Egyptian goose (*Alopochen aegyptiacus*) at the KVI Division of Avian Diseases. Previous reports on WNV detection in birds were from Eilat, Israel, which is an important bird migration site, and from the coastal plain of Israel [28]. The same work tested the exposure of storks to WNV, by means of serological response, demonstrating that migrating birds from different locations along the Great Rift Valley were exposed to the virus [28]. The recent report on WNV exposure in horses describes a somewhat similar picture, where positive animals were identified in the central coastal plain and in a few spots along the bird migration route [10]. The most recent study identified exposed domestic farm animals, including horses and donkeys, throughout the central and northern part of Israel, as well as the central part of the PA [26]. All the positive avian species and equine cases analysed in this report were from the central part of Israel, between the north of the Sharon region, and the central coastal plain. It is conceivable that migrating birds traveling along the Great Rift Valley, as well as horses living in the eastern part of the country, were also affected, but such cases have not been reported to the KVI. Four hooded crows have been found WNV-positive in this outbreak. Crows and corvids in general are highly susceptible to WNV infection, more than non-corvids [29]. Most of the other WNV-infected birds were a gull, geese and owls, species that are considered reservoirs of the virus that may transfer it further following their viremia [30]. Serological examination conducted during the 1960s suggested that the turtle dove (*Streptopelia turtur*) may play a role in WNV infection [31], but the findings reported herein may suggest that the species described above may currently be dominant as a reservoir.

The different viral load detected in the cerebellum and medulla of the horse labeled Eq142 (Table 3) may suggest that there is variability in the viral load in different regions of the brain, and multiple sites need to be examined, when attempting to detect WNV RNA in a *post-mortem* analysis. However, since this was found only in one animal, and since the different organs in the other horses generated similar Cq values in the RT-qPCR test, additional work is needed to firmly establish whether there are preferable regions in the brain for WNV molecular diagnosis.

The pathological findings of the suspected birds were characteristic of WNV infection, demonstrating the potential risk of virus spread to susceptible species, both domesticated and wildlife, as was reported in Israel during the early 2000s [12]. The neurological disorders observed reflected an acute state of inflammation, as was recently described in WNV infection [32]. The gross pathology of the brain and subsequent histological analysis of the owls, for example non-suppurative encephalitis with glial nodules and neuronal necrosis are characteristic of viral infection of the central nervous system, which is the hallmark of acute WNF in birds [33]. Conspicuous intraosseus hemorrhage, as was found in the long-eared owl AV156 and the hooded crow 1505, may result from trauma following collision with obstacles in their path of flight or loss of balance and falling, due to the neurological disease [17]. The RT-qPCR results in avian species showed medium-to-high viral load in visceral organs and in the brain. The relatively high viral load in cloacal samples may suggest that cloacal swabs should be used as a simple *ante-mortem* sampling method that does not require an invasive procedure and can provide a sensitive indication for the presence of the virus. Detecting the virus in blood of hooded crow AV169 reflects another means to detect the virus in live birds, at least during the viremia stage.

The last reports on acute WNF in horses in Israel were published in the early 2000s [18]. Although recent studies examined the seroprevalence in domesticated animals, especially horses, both in Israel and the PA [10, 26], the present study is the first to describe direct detection of WNV in horses during the last 17 years in Israel. The quantitative evaluation of the viral load in different organs of the same bird, and in the horse brain, show that during the time of sampling, the viral load varies between different organs. It may also suggest that cloacal swab sampling and ocular sampling provide good indication for viral infection, thereby obviating the need for visceral organ dissection. The successful culturing of four WNV-positive samples obtained in this study enables a more comprehensive genomic characterization and better understanding of the infection process of each strain, compared with previously studied strains. However, the very low viral load and poor condition of some of the samples was not sufficient for virus isolation, thereby limiting the number of successful isolates.

A simultaneous increase in WNF among humans occurred during the summer of 2018, resulting in 139 confirmed cases, of which 76 suffered from neurological complications and seven deceased [34]. The phylogenetic analysis of this outbreak showed that the infecting strain was WNV Lineage 1 clade 1a, Eastern-European and Mediterranean subtypes of Cluster 2. The phylogenetic distribution of the equid and avian samples showed clear distribution into three groups, with varying boostrap support. The grouping of five samples into Cluster 4 was supported by 85% bootstrap replications. Analysis of the Lineage 1a samples without Lineage 2 sequences somewhat increased the bootstrap support values, while retaining the same partition (Additional file 1: Figure S2). The partition between Cluster 2 Mediterranean (two samples) and Cluster 2 Eastern European (five samples from 2018 and one from 2016) relied on bootstrap support lower than 70%. However, the pattern generated by the annotated sequence grouping suggested that they were indeed part of the Med and EE groups, as shown in Fig. 5 and Additional file 1: Figure S2. The successful culturing of four samples allows further characterization of the new isolates by NGS and cell-based assays. The fact that out of 14 positive samples, only four were cultured demonstrates the difficulty in obtaining infectious virus from clinical samples on one hand, and the usefulness of RT-qPCR as a sensitive and specific surveillance tool.

Out of all the samples successfully sequenced, only two isolates (one horse and one domesticated goose) were of Cluster 2 Mediterranean subtype, which is the only cluster reported to circulate in mosquitoes and humans in Israel between 2004 and 2018 [21, 25]. Interestingly, four samples (horse, donkey and long-eared owls) were grouped with annotated Cluster 4 samples. To our knowledge, there were no confirmed reports on Cluster 4 WNV in Israel since 2004 (Fig. 5). It is conceivable that larger sequence length could have provided a better, more robust support for the subtype partitioning. Since the viral load in many of the samples was very low, it was not possible to obtain longer sequences. Nevertheless, complete genome analysis of the cultured strains is currently underway and is expected to provide a more comprehensive insight on the currently circulating WNV strains in Israel (manuscript in preparation).

The results of this study therefore highlight the importance of continuous WNV surveillance in equids and avian species, in order to complement the data obtained from mosquito sampling and human patient examination. This study also demonstrates the benefit of a combined approach to investigate WNV infection in wildlife and domestic animals, involving both pathological and molecular analyses.

## Conclusions

Constant surveillance of WNV in animals and birds is crucial to obtain a comprehensive view of its circulation, to complement the surveillance in humans and mosquitos. This study showed that at least three WNV strains were circulating in Israel during 2016 and 2018. Additionally, quantitative analysis of the positive samples suggested that there is large variation in the viral load and that cloacal swabs may be useful as a sampling method for testing suspected avian species.

## Supplementary information

**Additional file 1: Figure** **S1.** Generation of Standard curve of the NS2A test. **Alignment S1. **Sequences obtained in this study. **Figure S2. **Phylogenetic analysis of Lineage 1a including sequences obtained in this study.

## Data Availability

Data supporting the conclusions of this article are included within the article. The sequences obtained in this study are available in Additional file 1.

## Abbreviations

WNV: West Nile virus
RT-qPCR: Reverse transcription quantitative polymerase chain reaction
PM: *Post-mortem*
CNS: Central nervous system
Cq: Quantification cycle
