# Supplementary material for: Molecular characterization of the re-emerging West Nile virus in avian species and equids in Israel, 2018, and pathological description of the disease
Source: Parasit Vectors. 2020 Oct 22;13:528. doi: 10.1186/s13071-020-04399-2 (PMC7579921; doi:10.1186/s13071-020-04399-2)
Supplement: Supplementary file 1 — Additional file 1: Figure S1. Generation of Standard curve of the NS2A test. Alignment S1. Sequences obtained in this study. Figure S2. Phylogenetic analysis of Lineage 1a including sequences obtained in this study. [file 13071_2020_4399_MOESM1_ESM.docx]

**Molecular characterization of the re-emerging West Nile Virus in avian species and equids in Israel, 2018, and pathological description of the disease**

Gili Schvartz^1†^, Yigal Farnoushi^2†^, Asaf Berkowitz^2^, Nir Edery^3^ Shelly Hann^3^, Amir Steinman^4^, Avishai Lublin^2§^ and Oran Erster^1,5§^

^1^Division of Virology, Kimron Veterinary Institute, Bet Dagan, Israel

^2^Division of Avian diseases, Kimron Veterinary Institute, Bet Dagan, Israel

^3^ Division of Pathology, Kimron Veterinary Institute, Bet Dagan, Israel

^4^Koret School of Veterinary Medicine, The Robert H. Smith Faculty of Agriculture, Food and Environment, The Hebrew University of Jerusalem, Rehovot 7610001, Israel.

^5^Present address: Central Virology Laboratory, Israel Ministry of Health, Sheba Medical Center, Ramat Gan, Israel

^†^Gili Shvartz and Yigal Farnoushi contributed equally to this work

^§^Equal Senior Author

|  |
| --- |
| **Additional file 1: Figure S1** **Generation of Standard curve of the NS2A test.** NS2A PCR product at measured quantity was serially diluted and used for the qPCR test, to generate the curve and calculate the resulting formula that converts Cq values to copy number. The resulting calculation was then used to quantify the viral copy number in the examined samples. |

**Additional file 1: Alignment S1**

Sequences obtained in this work

>KVI_Egyptian goose Y8971 MK343716

TCGCATGCTCCCGGCCGCCTGGCGGCCGCGGGAATTCGATTCACAGCAATTGCTCCGACCCGAGCAGTGCTGGATCGATGGAGAGGTGTGAACAAACAAACAGCGATGAAACACCTTCTGAGTTTTAAGAAAGAACTAGGGAACTTGACCAGTGCTATCAATCGGCGGAGCTCAAAACAAAAGAAAAGAGGAGGAAATATTGGAATTGCAGCCATGATTGGCCTGATCGCCAGCGTAGGAGCAGTTACCCTCTCTAACTTCCAAGGGAAGGTTATGATGACGGTAAATGCTACTGACGTCACAGATGTCATCACGATTCCAACAGCTGCTGGAAAGAACCTATGCATTGTCAGAGCAATGGATGTAGGATACATGTGTGATGACACTATCACATATGAATGCCCAGTGCTGTCGGCTGGTAATGATCCAGAAGACATCGACTGTTGGTGCACAAAGTCAGCAGTCTACGTCGGGTATGGAAGATGCACCAAGACACGCCACTCAAGACGCAGTCGGAGGTCACTGACAGTGCAGACACACGGAGAAAGCACTCTAGCGAACAAAAAGGGGGCTTGGATGGACAGCACCAAGGCCACAAGGTATCTGGTGAAAACAGAATCATGGATCTTGAGGAACCCTGGATATGCCCTGGTGGCAGCTGTCATTGGTTGGATGCTTGGAAGCAACACCATGCAGCGAGTTGTGTTTGTCGTGCTGTTGCTCTTGGTGGCTCCAGCCTACAGCTTCAACTGCCTTGGAATGAGCAACAGAGACTTCTTGGAGGGAGTATCTGGAGCAACATGGGTGGATCTGGTTCTCGAAGGCGACAGCTGCGTGACTATCATGTCTAAGGACAAGCCTACCATTGATGTGAAGATGATGAATATGGAGGCTGCTAACCTGGCAGAGGTCCGCAGTTATTGCTATTTGGCTACCGTCAGCGATCTCTCCGCCAAAGCTGCATGCCCGACCATGGGAGAAGCTCACAATGACAAACGTGCTGACCCAGCTTTTGTGTGCAGACAAGGAGTGGTGGACAGGGGCTGGGGCAACGGCTGCGGACTATTTGGCAAAGGAAGCATTGACACATGCGCCAAATTTGCCTGCTCCACCAAGGCAACAGGAAGAACCATCTTGAAAGAGAATATCAAGTATGAAGTGGCCATCTTTGTCCATGGACCAACCACTGTGGAGTCGCATGGAAACTACTCCACACAGAATCACTAGTGAATTCGCGGCCGCCTGCAGGTCGACCATATGGGAGAGCNCCCAACGCGTGG

>KVI_horse Eq142

GCAGTTACCCTCTCTAACTTTCAAGGGAAAGTTATGATGACGGTAAACGCTACTGACGTCACAGATGTCATCACGATTCCAACAGCTGCTGGAAAGAATCTATGCATTGTCAGAGCAATGGATGTGGGATACATGTGTGATGACACTATCACCTATGAATGCCCAGTGCTGTCGGCCGGTAATGATCCAGAAGACATCGACTGTTGGTGCACAAAGTCAGCGGTCTACGTCAGGTATGGAAGATGCACCAAGACACGCCACTCAAGACGCAGTAGGAGGTCACTGACAGTGCAGACACACGGAGAAAGCACTCTAGCGAACAAAAAGGGGGCTTGGATGGACAGCACCAAGGCCACAAGGTATCTGGTAAAAACAGAATCATGGATCCTGAGGAACCCTGGATATGCCCTGGTGGCAGCTGTCATTGGTTGGATGCTTGGAAGCAACACCATGCAGCGAGTTGTGTTTGTCGTGTTATTGCTCCTGGTGGCTCCAGCCTACAGCTTCAACTGCCTTGGAATGAGCAACAGAGACTTCTTGGAGGGAGTATCTGGAGCAACATGGGTGGATTTGGTTCTCGAAGGCGACAGCTGCGTGACTATCATGTCCAAGGACAAGCCAACCATTGATGTGAAGATGATGAATATGGAGGCTGCCAACCTGGCAGAGGTCCGCAGTTATTGCTATTTGGCTACCGTCAGCGATCTCTCCACCAAAGCTGCATGCCCGACCATGGGAGAAGCTCACAACGACAAACGTGCTGACCCAGCTTTTGTGTGCAAACAAGGAGTGGTGGACAGGGGCTGGGGCAACGGCTGCGGACTATTTGGCAAAGGAAGCATTGACACATGCGCCAAATTTGCTTGCTCCACCAAGGCAACAGGAAGAACCATCTTGAAAGAGAACATCAAGTATAAGGGCCTCTTTTCCAKGACCAACCA

>KVI Av148 MK343718

CCGGGGCGGTCAAATATGCTAAACGCGGAATGCCCCGCGTGTTGTCCTTGATTGGACTGAAGAGGGCAATGTTGAGCCTGATTGACGGCAAGGGGCCAATACGATTCGTGTTGGCTCTCTTGGCGTTCTTCAGGTTCACAGCAATTGCTCCGACCCGAGCAGTGCTGGATCGATGGAGAGGTGTGAACAAACAAACAGCGATGAAACACCTTCTGAGTTTTAAGAAAGAACTAGGGACCTTGACCAGTGCTATCAATCGGCGGAGCTCAAAACAAAAGAAAAGAGGAGGAAATACTGGAATTGCAGCCATGATTGGCCTGATCGCCAGCGTAGGAGCAGTTACCCTCTCTAACTTCCAAGGGAAGGTTATGATGACGGTAAATGCTACTGACGTCACAGATGTCATCACGATTCCAACAGCTGCTGGAAAGAACCTATGCATTGTCAGAGCAATGGATGTAGGATACATGTGTGATGACACTATCACATATGAATGCCCAGTGCTGTCGGCCGGTAATGATCCAGAAGACATCGACTGTTGGTGCACAAAGTCAGCAGTCTACGTCAGGTATGGAAGATGCACCAAGACACGCCACTCAAGACGCAGTCGGAGGTCACTGACAGTGCAGACACACGGAGAAAGCACTCTAGCGAATAAAAAGGGTGCTTGGATGGACAGCACCAAGGCCACAAGGTATCTGGTGAAAACAGAATCATGGATCTTGAGGAACCCTGGATATGCCCTGGTGGCAGCTGTCATTGGTTGGATGCTTGGAAGCAACACCATGCAGCGAGTTGTGTTTGTCGTGCTGTTGCTCTTGGTGGCTCCAGCTTACAGCTTCAACTGCCTTGGAATGAGCAACAGA

>KVI_crow Y1459-3

GGTTCACAGCAATTGCTCCGACCCGAGCAGTGCTGGATCGATGGAGAGGTGTGAACAAACAAACAGCGATGAAACACCTTCTGAGTTTTAAGAAAGAACTAGGGACCTTGACCAGTGCTATCAATCGGCGGAGCTCAAAACAAAAGAAAAGAGGAGGAAATACTGGAATTGCAGCCATGATTGGCCTGATCGCCAGCGTAGGAGCAGTTACCCTCTCTAACTTCCAAGGGAAGGTTATGATGACGGTAAATGCTACTGACGTCACAGATGTCATCACGATTCCAACAGCTGCTGGAAAGAACCTATGCATTGTCAGAGCAATGGATGTAGGATACATGTGTGATGACACTATCACATATGAATGCCCAGTGCTGTCGGCCGGTAATGATCCAGAAGACATCGACTGTTGGTGCACAAAGTCAGCAGTCTACGTCAGGTATGGAAGATGCACCAAGACACGCCACTCAAGACGCAGTCGGAGGTCACTGACAGTGCAGACACACGGAGAAAGCACTCTAGCGAATAAAAAGGGTGCTTGGATGGACAGCACCAAGGCCACAAGGTATCTGGTGAAAACAGAATCATGGATCTTGAGGAACCCTGGATATGCCCTGGTGGCAGCTGTCATTGGTTGGATGCTTGGAAGCAACACCATGCAGCGAGTTGTGTTTGTCGTGCTGTTGCTCTTGGTGGCTCCAGCTTACAGCTT

>KVI_crow Y1505 MK343719

TGTCAATATGCTAAAACGCGGAATGCCCCGCGTGTGTCCTTGATTGGACTGAAGAGGGCAATGTTGAGCCTGATTGACGGCAAGGGGCCNATACGATTCGTGTTGGCTCTCTTGGCGTTCTTCAGGTTCACAGCAATTGCTCCGACCCGAGCAGTGCTGGATCGATGGAGAGGTGTGAACAAACAAACAGCGATGAAACACCTTCTGAGTTTTAAGAAAGAACTAGGGACCTTGACCAGTGCTATCAATCGGCGGAGCTCAAAACAAAAGAAAAGAGGAGGAAATACTGGAATTGCAGCCATGATTGGCCTGATCGCCAGCGTAGGAGCAGTTACCCTCTCTAACTTCCAAGGGAAGGTTATGATGACGGTAAATGCTACTGACGTCACAGATGTCATCACGATTCCAACAGCTGCTGGAAAGAACCTATGCATTGTCAGAGCAATGGATGTAGGATACATGTGTGATGACACTATCACATATGAATGCCCAGTGCTGTCGGCCGGTAATGATCCAGAAGACATCGACTGTTGGTGCACAAAGTCAGCAGTCTACGTCAGGTATGGAAGATGCACCAAGACACGCCACTCAAGACGCAGTCGGAGGTCACTGACAGTGCAGACACACGGAGAAAGCACTCTAGCGAATAAAAAGGGTGCTTGGATGGACAGCACCAAGGCCACAAGGTATCTGGTGAAAACAGAATCATGGATCTTGAGGAACCCTGGATATGCCCTGGTGGCAGCTGTCATTGGGTGGATGCTTGGAAGCAACACCATGCAGCGAGTTGTGTTTGTCGTGCTGTTGCTCTTGGTGGCTCCAGCTTACAGCTTCAACTGCCTTGGAATGAGCAACAGAGACTTCCTGGAGGGAGTGTCCGGGAGC

>KVI_crow Y1514

CTGTCAATATGCTAAAACGCGGAATGCCCCGCGTGTTGTCTTTGATTGGACTGAAGAGGGCAATGTTGAGCCTGATTGACGGCAAGGGGCCAATACGATTCGTGTTGGCTCTCTTGGCGTTCTTCAGGTTCACAGCAATTGCTCCGACCCGAGCAGTGCTGGATCGATGGAGAGGTGTGAACAAACAAACAGCGATGAAACACCTTCTGAGTTTTAAGAAAGAACTAGGGACCTTGACCAGTGCTATCAATCGGCGGAGCTCAAAACAAAAGAAAAGAGGAGGAAATACTGGAATTGCAGCCATGATTGGCCTGATCGCCAGCGTAGGAGCAGTTACCCTCTCTAACTTCCAAGGGAAGGTTATGATGACGGTAAATGCTACTGACGTCACAGATGTCATCACGATTCCAACAGCTGCTGGAAAGAACCTATGCATTGTCAGAGCAATGGATGTAGGATACATGTGTGATGACACTATCACATATGAATGCCCAGTGCTGTCGGCCGGTAATGATCCAGAAGACATCGACTGTTGGTGCACAAAGTCAGCAGTCTACGTCAGGTATGGAAGATGCACCAAGACACGCCACTCAAGACGCAGTCGGAGGTCACTGACAGTGCAGACACACGGAGAAAGCACTCTAGCGAATAAAAAGGGTGCTTGGATGGACAGCACCAAGGCCACAAGGTATCTGGTGAAAACAGAATCATGGATCTTGAGGAACCCTGGATATGCCCTGGTGGCAGCTGTCATTGGGTGGATGCTTGGAAGCAACACCATGCAGCGAGTTGTGTTTGTCGTGCTGTTGCTCTTGGTGGCTCCAGCTTACAGCTTCAACTGCCTTGGAATGAGCAACAGAGACTTCCTGGAGGGAGTGTCCGGAGCA

>KVI_goose Av152

GTATGCCCCGCGTGTTGTCCTTGATTGGACGGATGAGGGCAATGTTGAGCCTGATTGACGGCAAGGGGCCAATACGATTCGTGTTGGCTCTCTTGGCGTTCTTCAGGTTCACAGCAATTGCTCCGACCCGAGCAGTGCTGGATCGATGGAGAGGTGTGAACAAACAAACAGCGATGAAACACCTTCTGAGTTTTAAGAAAGAACTAGGGACCTTGACCAGTGCTATCAATCGGCGGAGCTCAAAACAAAAGAAAAGAGGAGGAAATACTGGAATTGCAGCCATGATTGGCCTGATCGCCAGCGTAGGAGCAGTTACCCTCTCTAACTTCCAAGGGAAGGTTATGATGACGGTAAATGCTACTGACGTCACAGATGTCATCACGATTCCAACAGCTGCTGGAAAGAACCTATGCATTGTCAGAGCAATGGATGTAGGATACATGTGTGATGACACTATCACATATGAATGCCCAGTGCTGTCGGCYGGTAATGATCCAGAAGACATCGACTGTTGGTGCACAAAGTCAGCAGTCTACGTCAGGTATGGAAGATGCACCAAGACACGCCACTCAAGACGCAGTCGGAGGTCACTGACAGTGCAGACACACGGAGAAAGCACTCTAGCGAATAAAAAGGGTGCTTGGATGGACAGCACCAAGGCCACAAGGTATCTGGTGAAAACAGAATCATGGATCTTGAGGAACCCTGGATATGCCCTGGTGGCAGCTGTCATTGGTTGGATGCTTGGAAGCAACACCATGCAGCGAGTTGTGTTTGTCGTGCTGTTGCTCTTGGTGGCTCCAGCTTACAGCTTCAACTGCCTTGGAATGAGCAACAGAGACTTCCTGGAGGGAGT

>KVI_goose Av153

CAATGCCCCGCGTATTGTCCTTGATTGGACTGAAGAGGGCAATGTTGAGCCTGATTGACGGTAGGGGGCCAATACGATTCGTGTTGGCTCTCTTGGCGTTCTTCAGGTTCACAGCAATTGCTCCGACTCGAGCAGTGCTGGATCGATGGAGAGGTGTGAACAAACAAACAGCGATGAAACACCTTCTGAGTTTTAAGAAAGAACTAGGGACCTTGACCAGTGCTATCAATCGGCGGAGCTCAAAACAAAAGAAAAGAGGAGGAAAGACCGGAATTGCAGTCATGATTGGCCTTATCGCCAGCGTAGGAGCAGTTACCCTCTCTAACTTTCAAGGGAAAGTTATGATGACGGTAAACGCTACTGACGTCACAGATGTCATCACGATTCCAACAGCTGCTGGAAAGAATCTATGCATTGTCAGAGCAATGGATGTGGGATACATGTGTGATGACACTATCACCTATGAATGCCCAGTGCTGTCGGCCGGTAATGATCCAGAAGACATCGACTGTTGGTGCACAAAGTCAGCGGTCTACGTCAGGTATGGAAGATGCACCAAGACACGCCACTCAAGACGCAGTAGGAGGTCACTGACAGTGCAGACACACGGAGAAAGCACTCTAGCGAACAAAAAGGGGGCTTGGATGGACAGCACCAAGGCCACAAGGTATCTGGTAAAAACAGAATCATGGATCCTGAGGAACCCTGGATATGCCCTGGTGGCAGCTGTCATTGGTTGGATGCTTGGAAGCAACACCATGCAGCGAGTTGTGTTTGTCGTGTTATTGCTCCTGGTGGCTCCAGCCTACAGCTTCAACTGCCTTGGAATGAGCAACAGAGACTTCCTGGAGGGA

>KVI_owl Av156

GGTTCACAGCAATTGCTCCGACCCGAGCAGTGCTGGATCGATGGAGAGGTGTGAACAAACAAACAGCGATGAAACACCTTCTGAGTTTTAAGAAGGAACTAGGGACCTTGACCAGTGCTATCAATCGGCGGAGCTCAAAACAAAAGAAAAGAGGAGGAAAGACCGGAATTGCAGTCATGATTGGCCTGATCGCCAGCGTAGGAGCAGTTACCCTCTCTAACTTTCAAGGGAAGGTGATGATGACGGTAAATGCTACTGACGTCACAGATGTCATCACGATTCCAACAGCTGCTGGAAAGAACCTATGCATTGTCAGAGCAATGGATGTGGGATACATGTGCGATGATACTATCACTTATGAATGCCCAGTGCTGTCGGCTGGTAATGATCCAGAAGACATCGACTGCTGGTGCACAAAGTCAGCAGTCTACGTCAGGTATGGAAGATGCACCAAGACACGCCACTCAAGACGCAGTCGGAGGTCACTGACAGTGCAGACACACGGAGAAAGCACTCTAGCGAACAAGAAGGGGGCTTGGATGGACAGCACCAAGGCCACAAGGTATTTGGTAAAAACAGAATCATGGATCTTGAGGAACCCTGGATATGCCCTGGTGGCAGCCGTCATTGGTTGGATGCTTGGGAGCAACACCATGCAGAGAGTTGTGTTTGTCGTGCTATTGCTTTTGGTGGCCCCAGCTTACAGCTT

>KVI_owl Av157

CAATATGCTAAAACGCGGTATGCCCCGCGTGTTGTCCTTGATTGGACTGAAGAGGGCTATGTTGAGCCTGATCGACGGCAAGGGGCCAATACGATTTGTGTTGGCTCTCTTGGCGTTCTTCAGGTTCACAGCAATTGCTCCGACCCGAGCAGTGCTGGATCGATGGAGAGGTGTGAACAAACAAACAGCGATGAAACACCTTCTGAGTTTTAAGAAGGAACTAGGGACCTTGACCAGTGCTATCAATCGGCGGAGCTCAAAACAAAAGAAAAGAGGAGGAAAGACCGGAATTGCAGTCATGATTGGCCTGATCGCCAGCGTAGGAGCAGTTACCCTCTCTAACTTTCAAGGGAAGGTGATGATGACGGTAAATGCTACTGACGTCACAGATGTCATCACGATTCCAACAGCTGCTGGAAAGAACCTATGCATTGTCAGAGCAATGGATGTGGGATACATGTGCGATGATACTATCACTTATGAATGCCCAGTGCTGTCGGCTGGTAATGATCCAGAAGACATCGACTGCTGGTGCACAAAGTCAGCAGTCTACGTCAGGTATGGAAGATGCACCAAGACACGCCACTCAAGACGCAGTCGGAGGTCACTGACAGTGCAGACACACGGAGAAAGCACTCTAGCGAACAAGAAGGGGGCTTGGATGGACAGCACCAAGGCCACAAGGTATTTGGTAAAAACAGAATCATGGATCTTGAGGAACCCTGGATATGCCCTGGTGGCAGCCGTCATTGGTTGGATGCTTGGGAGCAACACCATGCAGAGAGTTGTGTTTGTCGTGCTATTGCTTTTTGGTGGCCCCCAGCTTACAGCTTCAACTGCCTTGGAATGAGCAACAGAGACTTTCCCTGGAAGGGGAGTC

>KVI_crow Av169C

GATGCCCCGCGTGTTGTCCTTGATTGGACTGAAGAGGGCAATGTTGAGCCTGATTGACGGCAAGGGGCCAATACGATTCGTGTTGGCTCTCTTGGCGTTCTTCAGGTTCACAGCAATTGCTCCGACCCGAGCAGTGCTGGATCGATGGAGAGGTGTGAACAAACAAACAGCGATGAAACACCTTCTGAGTTTTAAGAAAGAACTAGGGACCTTGACCAGTGCTATCAATCGGCGGAGCTCAAAACAAAAGAAAAGAGGAGGAAATACTGGAATTGCAGCCATGATTGGCCTGATCGCCAGCGTAGGAGCAGTTACCCTCTCTAACTTCCAAGGGAAGGTTATGATGACGGTAAATGCTACTGACGTCACAGATGTCATCACGATTCCAACAGCTGCTGGAAAGAACCTATGCATTGTCAGAGCAATGGATGTAGGATACATGTGTGATGACACTATCACATATGAATGCCCAGTGCTGTCGGCCGGTAATGATCCAGAAGACATCGACTGTTGGTGCACAAAGTCAGCAGTCTACGTCAGGTATGGAAGATGCACCAAGACACGCCACTCAAGACGCAGTCGGAGGTCACTGACAGTGCAGACACACGGAGAAAGCACTCTAGCGAATAAAAAGGGTGCTTGGATGGACAGCACCAAGGCCACAAGGTATCTGGTGAAAACAGAATCATGGATCTTGAGGAACCCTGGATATGCCCTGGTGGCAGCTGTCATTGGTTGGATGCTTGGAAGCAACACCATGCAGCGAGTTGTGTTTGTCGTGCTGTTGCTCTTGGTGGCTCCAGCTTACAGCTTCAACTGCCTTGGAATGAGCAACAGAGACTTCCTGGAGGAGT

>KVI_horse Eq111

CTGTCAATATGCTAAAAAACGCGGTATGCCCCCGCGTGTTGTCCTTGATTGGACTGAAGAGGGGCTATGTTGAGCCTGATCGACGGCAAGGGGCCAATACGATTTGTGTTGGCTCTCTTGGCGTTCTTCAGGTTCACAGCAATTGCTCCGACCCGAGCAGTGCTGGATCGATGGAGAGGTGTGAACAAACAAACAGCGATGAAACACCTTCTGAGTTTTAAGAAGGAACTAGGGACCTTGACCAGTGCTATCAATCGGCGGAGCTCAAAACAAAAGAAAAGAGGAGGAAAGACCGGAATTGCAGTCATGATTGGCCTGATCGCCAGCGTAGGAGCAGTTACCCTCTCTAACTTTCAAGGGAAGGTGATGATGACGGTAAATGCTACTGACGTCACAGATGTCATCACGATTCCAACAGCTGCTGGAAAGAACCTATGCATTGTCAGAGCAATGGATGTGGGATACATGTGCGATGATACTATCACTTATGAATGCCCAGTGCTGTCGGCTGGTAATGATCCAGAAGACATCGACTGCTGGTGCACAAAGTCAGCAGTCTACGTCAGGTATGGAAGATGCACCAAGACACGCCACTCAAGACGCAGTCGGAGGTCACTGACAGTGCAGACACACGGAGAAAGCACTCTAGCGAACAAGAAGGGGGCTTGGATGGACAGCACCAAGGCCACAAGGTATTTGGTAAAAACAGAATCATGGATCTTGAGGAACCCTGGATATGCCCTGGTGGCAGCCGTCATTGGTTGGATGCTTGGGAGCAACACCATGCAGAGAGTTGTGTTTGTCGTGCTATTGCTTTTGGTGGCCCCAGCTTACAGCTTCAACTGCCTTGGAATGAGCAACAGA

>KVI_donkey Eq115

GGTTCACAGCAATTGCTCCGACCCGAGCAGTGCTGGATCGATGGAGAGGTGTGAACAAACAAACAGCGATGAAACACCTTCTGAGTTTTAAGAAGGAACTAGGGACCTTGACCAGTGCTATCAATCGGCGGAGCTCAAAACAAAAGAAAAGAGGAGGAAAGACCGGAATTGCAGTCATGATTGGCCTGATCGCCAGCGTAGGAGCAGTTACCCTCTCTAACTTTCAAGGGAAGGTGATGATGACGGTAAATGCTACTGACGTCACAGATGTCATCACGATTCCAACAGCTGCTGGAAAGAACCTATGCATTGTCAGAGCAATGGATGTGGGATACATGTGCGATGATACTATCACTTATGAATGCCCAGTGCTGTCGGCTGGTAATGATCCAGAAGACATCGACTGCTGGTGCACAAAGTCAGCAGTCTACGTCAGGTATGGAAGATGCACCAAGACACGCCACTCAAGACGCAGTCGGAGGTCACTGACAGTGCAGACACACGGAGAAAGCACTCTAGCGAACAAGAAGGGGGCTTGGATGGACAGCACCAAGGCCACAAGGTATTTGGTAAAAACAGAATCATGGATCTTGAGGAACCCTGGATATGCCCTGGTGGCAGCCGTCATTGGTTGGATGCTTGGGAGCAACACCATGCAGAGAGTTGTGTTTGTCGTGCTATTGCTTTTTGGTGGCCCCCAGCTTACAGCTT

|  |
| --- |
| **Additional file 1: Figure S2. Phylogenetic analysis of Lineage 1a.** Annotated West Nile virus (WNV) Lineage 1 strain sequences and samples obtained in this study were aligned and used to construct a phylogenetic dendrogram. The same samples and construction terms that were used for the analysis described in Figure 5 of the printer version were used here, excluding the Lineage 2 samples. The partition was supported by 1000 Bootstrap replications. Support values above 70% were designated near the branch to which they pertain.  Samples obtained in this study are labeled with rectangles. Arrows indicate Lineage 1 clusters of which single sequences are available in the GenBank. |
